# Specificity of Interactions between Components of Two Zinc ABC Transporters in *Paracoccus denitrificans*

**DOI:** 10.3390/ijms21239098

**Published:** 2020-11-30

**Authors:** Ady Berenice Meléndez, Daniel Valencia, Erik Thomas Yukl

**Affiliations:** Department of Chemistry and Biochemistry, New Mexico State University, Las Cruces, NM 88003, USA; adymel@nmsu.edu (A.B.M.); dwv8@nmsu.edu (D.V.)

**Keywords:** ABC transporters, solute-binding protein, zinc

## Abstract

Bacterial ATP binding cassette (ABC) transporters mediate the influx of numerous substrates. The cluster A-I ABC transporters are responsible for the specific uptake of the essential metals zinc, manganese or iron, making them necessary for survival in metal-limited environments, which for pathogens include the animal host. In *Paracoccus denitrificans*, there are two zinc ABC transporter systems: ZnuABC and AztABCD with apparently redundant functions under zinc-limited conditions. The unusual presence of two zinc ABC transporter systems in the same organism allowed for the investigation of specificity in the interaction between the solute binding protein (SBP) and its cognate permease. We also assessed the role of flexible loop features in the SBP in permease binding and zinc transport. The results indicate that the SBP–permease interaction is highly specific and does not require the flexible loop features of the SBP. We also present an expanded table of the properties of characterized cluster A-I SBPs and a multiple sequence alignment highlighting the conserved features. Through this analysis, an apparently new family of binding proteins associated with ABC transporters was identified. The presence of homologues in several human pathogens raises the possibility of using it as a target for the development of new antimicrobial therapies.

## 1. Introduction

The ATP binding cassette (ABC) transporters are a superfamily of membrane transport proteins with representatives across all kingdoms of life [1]. These systems are minimally composed of a membrane spanning permease formed by two transmembrane domains or subunits and an intracellular ATPase formed by two nucleotide binding domains or subunits. The former provides a pathway for the translocation of a vast variety of substrates across the cytoplasmic membrane while the latter powers their active transport by ATP hydrolysis. In prokaryotic importers of this type, an extracellular solute binding protein (SBP) component is typically required [2], which may be periplasmic, membrane tethered or fused to the permease [3]. The function of the SBP is to bind the substrate with a high affinity and specificity and to deliver it to the permease for import into the cytoplasm [4].

Generally, prokaryotic ABC transporters are expressed from operons encoding all the necessary components, including a single SBP that interacts specifically with its cognate permease. However, there are examples where a single permease can interact with multiple SBPs, either encoded within the operon as in the case of the amino acid transporter HisQMP [5] or with so-called “orphan” SBPs encoded elsewhere in the genome [6,7]. Through the construction of hybrid transporter systems, it was recently shown that the SBP for one ABC transporter could facilitate transport through a related transporter, thereby altering its substrate specificity [8]. To our knowledge, this is the only example of SBPs that can interact with multiple permeases, suggesting that, in most cases, the permease-SBP interaction is highly specific.

The substrate binding site for the SBP lies between two structurally related α/β domains. These proteins have been classified into 7 clusters (A–G) [9,10] according to the nature of the interdomain linker with further subdivisions based on substrate specificity. By this system, the presence of a long α-helix as the interdomain linker identifies proteins of cluster A-I, which directly bind zinc, manganese or iron. Phylogenetic analysis further divides cluster A-I proteins into three main groups [11]. Groups I and II are found in gram-positive and gram-negative bacteria, respectively. Both are specific for zinc and bind the metal with a 3His/1Glu or 3His/1H_2_O coordination environment. Both groups also contain a flexible loop near the high-affinity binding site that differs in length and composition. Group II loops are long and rich in His residues while Group I proteins have loops that tend to be shorter and more variable in sequence. Finally, Group III is usually specific for manganese or iron, and coordinates metal through a 2His/1Asp/1Glu or 3His/1Asp ligand set and has no flexible loop.

These systems have attracted considerable attention as they are critical for survival in metal-limited environments such as the human host [12], making them attractive as potential antibiotic drug targets. In some organisms, a single cluster A-I SBP and its associated transporter system is critical for zinc import and virulence [13,14,15,16,17]. As previously mentioned, it is also possible for multiple zinc SBPs, including orphans expressed outside the ABC transporter operon, to serve the same permease/ATPase. This is particularly prevalent in *Streptococcus* species [7,18,19,20,21]. On the other hand, the expression of more than one intact zinc ABC transporter seems quite rare. *Listeria monocytogenes* encodes one complete zinc ABC transporter system (ZinABC) and another lacking an SBP (ZurAM), which appear to serve redundant roles in zinc acquisition [22]. The observation that ZurAM can function in the absence of ZinABC suggests that it may be served by an orphan SBP. Indeed, an orphan group I homologue (Uniprot ID: lmo1671) may serve this purpose, although its function remains uncharacterized. *Vibrio cholerae* encodes two apparently complete ABC transporters that function in zinc uptake [23], although one of these encodes a very unusual truncated SBP (ZrgA) whose independent function was not assessed.

**Figure 1:**
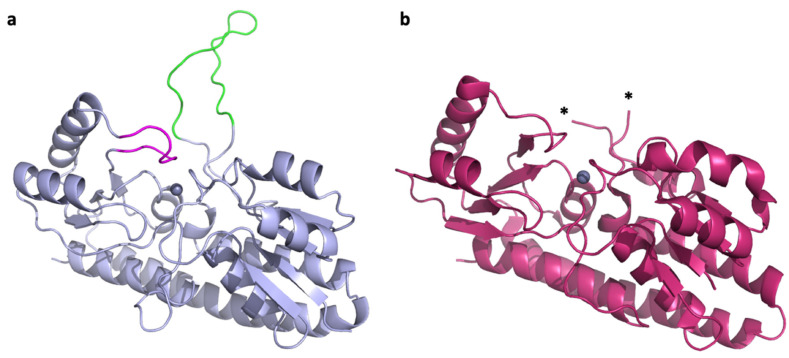
Zinc SBPs in *P. denitrificans*. (**a**) Crystal structure of *P. denitrificans* AztC (PDB ID: 5W57) [28] showing the D-Loop (green) and Z-Loop (magenta) structures. (**b**) Crystal structure of *E. coli* ZnuA (PDB ID: 2OSV) [29]. The D-Loop structure is not observed due to disorder and asterisks (*) mark the last modeled residues.

*Paracoccus denitrificans* is a model organism for the study of complex zinc import through ABC transporters. It encodes two intact zinc ABC transporter systems, AztABCD and ZnuABC. Both are regulated by the zinc-dependent transcriptional repressor Zur, which also regulates its own expression, and serve apparently redundant functions in zinc import [24,25,26]. Homologues of both systems are found in human pathogens *Citrobacter koseri* and in some strains of *Klebsiella pneumoniae* but were only characterized in the plant pathogen *Agrobacterium tumefaciens,* where both were also found to function in zinc import [27]. The SBPs for the *P. denitrificans* systems are AztC and ZnuA, which cluster with Group III and Group II subdivisions of the cluster A-I SBPs, respectively. Although Group III is typically associated with iron and manganese specificity, AztC binds zinc specifically in vitro [24]. It possesses a relatively short flexible loop with several His residues (hereafter referred to as the D-Loop), as well as a shorter loop feature that closes down over the zinc binding site (hereafter referred to as the Z-Loop) (Figure 1a). Thus, it shares characteristics in common with Group II and III. The structure from *P. denitrificans* ZnuA has not been experimentally determined, but it is 30% identical to that of *E. coli* (Figure 1b) where the flexible loop is not observed due to disorder. In fact, to our knowledge, no flexible loop structure has been observed in crystal structures for Group II proteins. The flexible loop in *P. denitrificans* ZnuA is extremely long (over 30 residues) and His-rich, making this a significant difference from AztC, with which it otherwise shares significant structural similarities as do all cluster A-I SBPs. As such, *P. denitrificans* provides an excellent opportunity to assess the specificity of the interaction between zinc SBPs and their cognate permeases and the role that the flexible loops play in this interaction. This is potentially important for drug development, as a promiscuous SBP-permease interaction will likely be a more challenging drug target.

In this article, the ability of AztC and ZnuA to crosstalk with permeases from the other system and the relevance of the loop structures for the acquisition and transfer of zinc in vivo is explored. We show that although the *P. denitrificans* AztABCD and ZnuABC systems are redundant under zinc depleted conditions, their respective SBPs cannot transfer zinc to the permease of the other system. We also found that mutations of the histidine rich loop structures did not affect the ability of *P. denitrificans* to acquire zinc from the environment, though there was a slight growth defect for the D-Loop deletion mutant of AztC. Thus, the functions of these structures remain somewhat enigmatic. Finally, an updated description of the currently characterized cluster A-I SBPs is presented that leads to the identification of what may be a novel family of metal transporting SBPs that function through ABC transporters.

## 2. Results

### 2.1. Zinc Transport in Hybrid znu and azt Transporter Systems

In order to test the specificity of the interaction between a zinc SBP and its cognate permease, *P. denitrificans* mutant strains were constructed with “hybrid” ABC transporters. In-frame, unmarked deletions were made such that these mutants encode only the SBP from one system (e.g., *znuA*), and the permease and ATPase from the other system (e.g., *aztAB*).

**Figure 2:**
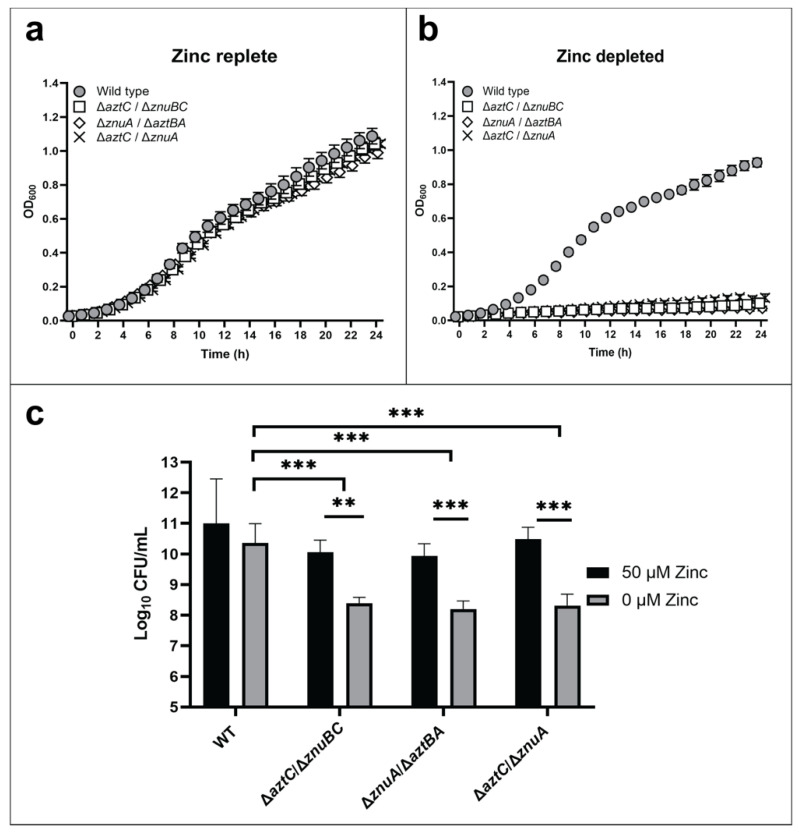
The ZnuABC and AztABCD transporter systems do not crosstalk. Growth curves at 600 nm in zinc-replete (**a**) or zinc-depleted (**b**) conditions for 24 h. Cellular viability quantification after 48 h of incubation (**c**). Error bars represent the mean ± S.D. of triplicate experiments. For the CFU, two-way ANOVA was used followed by Tukey’s multiple comparison test to show that viability was significantly decreased in mutant strains under zinc limited conditions, *** *p* < 0.001, ** *p* < 0.01.

Growth assays were conducted for wild type and mutant strains including a Δ*aztC/*Δ*znuA* double mutant previously shown to be deficient in zinc acquisition [26]. All strains grew comparably in zinc-replete media (Figure 2a). However, all “hybrid transporter” mutant strains exhibited a significantly reduced growth when compared to WT (Figure 2b) in zinc-limited media comparable to the double mutant. Cell viability after 48 h as determined by colony forming units (CFU) was also decreased under zinc-limited conditions for the mutants (Figure 2c). RT-PCR was used to confirm normal expression of the remaining ABC transporter genes, indicating that the phenotypes were not a result of polar effects influencing gene expression (Supplementary Figure S1). The observation that zinc supplementation rescues all mutant phenotypes further indicates that no significant secondary mutations occurred.

### 2.2. Flexible Loop Structures Are Not Essential for Zinc Transfer

It was previously demonstrated in vitro that deletion of the flexible loop from either ZnuA [26] or AztC [28] did not inhibit high-affinity zinc binding. In ZnuA, deletion did eliminate several additional, lower affinity binding sites whereas in AztC it eliminated interaction with the metallochaperone AztD. In order to evaluate the role of the SBP flexible loop, mutants were generated encoding a loop-deletion of one SBP (ΔLoop *aztC*) in the background of the null mutation of the other (Δ*znuA*).

**Figure 3:**
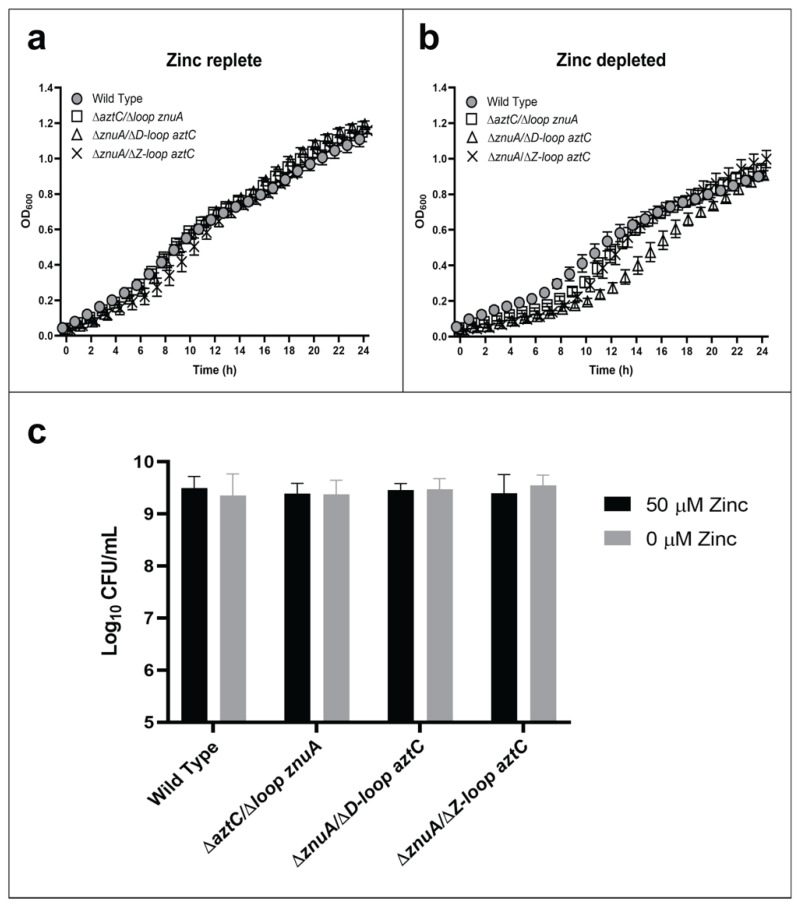
Flexible loop structures are not essential for zinc transport. Growth curves at 600 nm in zinc-replete (**a**) or zinc-depleted (**b**) conditions for 24 h. Cellular viability quantification after 48 h of incubation (**c**). Error bars represent the mean ± S.D. of triplicate experiments. For the CFU, two-way ANOVA was used followed by Tukey’s multiple comparison test to show that there was no statistically significant difference in viability between the mutants and WT.

In this case, only the mutant lacking the flexible loop of AztC (ΔD-Loop *aztC*) exhibited any growth defect in zinc-depleted media, and this was relatively minor (Figure 3). We also analyzed the role of another flexible loop feature present only in AztC (the so-called “Z-loop”), which closes down over the zinc site (Figure 1a) and regulates the rate of zinc dissociation [30]. It seemed likely that this feature may have a role in recognizing the permease. However, this mutant also exhibited no decrease in growth in zinc-limited media relative to WT. None of the mutants were significantly different from WT in terms of long-term viability as determined by CFU.

**Figure 4:**
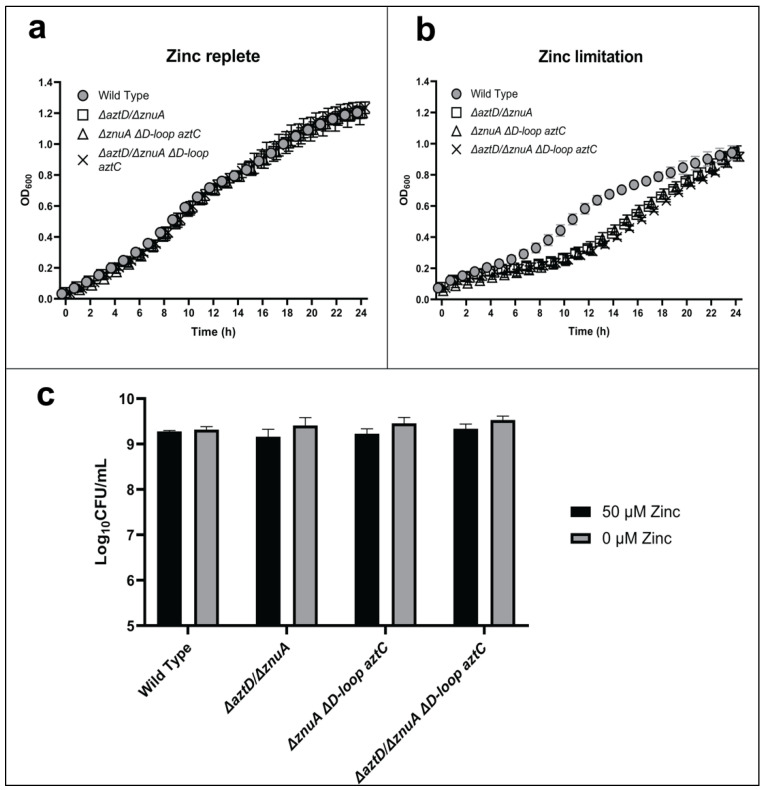
The role of AztD and the flexible loop of AztC in zinc transport. Growth curves at 600 nm in zinc-replete (**a**) or zinc-depleted (**b**) conditions for 24 h. Cellular viability quantification after 48 h of incubation (**c**). Error bars represent the mean ± S.D. of triplicate experiments. For the CFU, two-way ANOVA was used followed by Tukey’s multiple comparison test to show there was no statistically significant difference in viability between the mutants and WT.

We also investigated the function of the AztC D-Loop in conjunction with the metal chaperone AztD (Figure 4). Direct zinc transfer from AztD to AztC has been observed in vitro, and this process requires the AztC D-Loop [28]. Previous metal accumulation analysis of mutant strains further demonstrated that the presence of AztD led to WT levels of zinc accumulation but could not support growth in the absence of the SBPs in zinc-limited media [26]. This suggested that AztD could accumulate zinc in the periplasm but could not mediate transport into the cell in the absence of AztC. Each of the mutants lacking both *aztD* and *znuA* exhibited comparable, modest lags in growth and no difference in long-term viability, irrespective of the presence of the AztC D-Loop. Again, the phenotypes were rescued by addition of zinc. These results indicate that optimal zinc import through the AztABCD system requires both the AztD and the D-Loop of AztC, consistent with in vitro results.

### 2.3. Cluster A-I SBP Homology

Supplementary Table S1 shows an updated summary of those cluster A-I SBPs for which crystal structures exist or whose function has been characterized. The multiple sequence alignment (Supplementary Figure S2) demonstrates that all share recognizable features of cluster A-I SBPs including conservation of zinc ligands and an absolutely conserved Trp with the exception of ZrgA (VC2552), discussed below. As mentioned above, *P. denitrificans* ZnuA and AztC are in cluster A-I Groups II and III, respectively, with 25% sequence identity and 55% similarity. The results above confirm that they do not share permeases. To our knowledge, all instances of multiple SBPs serving the same permease come from within Group I, which share a greater degree of sequence homology (e.g., AdcA and AdcAII from S. pneumoniae are 39% and 73% identical and similar, respectively). Thus, the observation that AztC and ZnuA cannot interact with the permease from the other system may be a simple consequence of their divergence coupled with the fact that their permeases are similarly divergent with 26% and 58% sequence identity and similarity, respectively.

### 2.4. The ZrgA-Like Proteins

**Figure 5:**
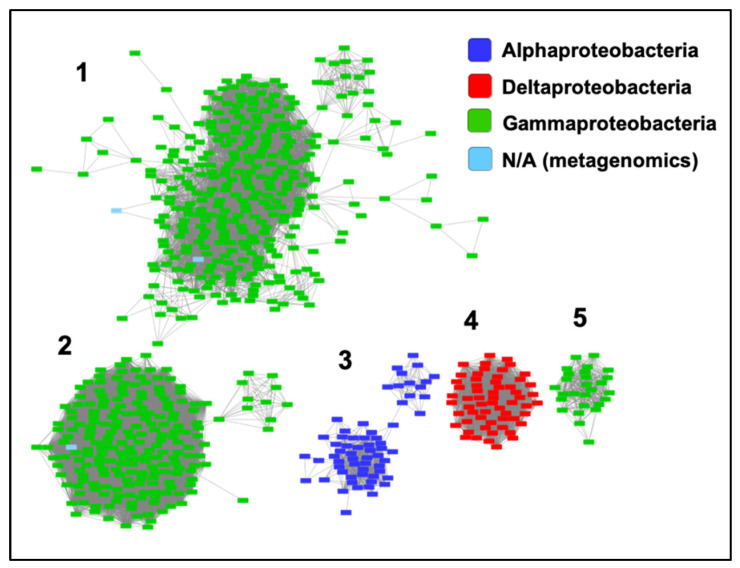
Relationships between ZrgA sequences. Sequence similarity network including 677 sequences filtered such that only the edges associated with E-values less than 10^−40^ are included in the network. Sequences are represented by rectangles colored according to class. The five largest clusters are indicated by numbers (1,2,3,4,5), which refer to the clusters analyzed in Supplementary Figure S3.

We have included ZrgA from *V. cholerae* in Supplementary Table S1 because it was identified as a zinc-specific binding protein for an ABC transporter in that organism [23]. However, ZrgA aligns only very poorly with the N-terminal domain of other cluster A-I proteins. Furthermore, it is the only sequence in which the metal ligands and Trp residue are not conserved as they are for all other cluster A-I sequences (Supplementary Figure S1). This protein also possesses an extremely long (~60 residues), apparently disordered region composed almost entirely of His, Glu, Asp, and Lys residues. Thus, it is probably inappropriate to classify this as a classical SBP, and conserved domain databases identify it only as a domain of unknown function. A BLASTP search of the UniProtKB database identified over 1000 ZrgA-like sequences with E values below 10^−20^ from various bacterial taxa. A sequence similarity network (Figure 5) shows that most ZrgA sequences cluster into five main groups according to bacterial class. This network was further analyzed to determine the conservation of genome neighborhoods within and between different clusters (Supplementary Figure S3). In total, 97% of these *zrgA* sequences are within five genes of ABC transporter genes with a median distance of 1 (adjacent), confirming their likely function as binding proteins for these transporters.

The largest ZrgA cluster (Group 1) is composed of sequences deriving almost entirely from various *Vibrio* species while the second largest cluster is almost entirely from the genus *Pseudomonas*, including multiple strains of the human pathogen *P. aeruginosa*. Group 3 is composed of sequences for various alphaproteobacteria. Group 4 are betaproteobacterial sequences with the majority deriving from the genus *Variovorax*. Finally, most Group 5 sequences are from the genus *Aeromonas*, which includes a number of emerging human pathogens [31].

## 3. Discussion

All of the data collected to date suggest that the interactions between an SBP and its cognate permease are exquisitely specific. Our data, as well as a previous study with *S. enterica* ZnuA [32], suggest that the SBP flexible loop structures are not essential for interaction with the permease and metal transport. Thus, the precise structural determinants of this specificity will likely have to await high-resolution structural data like the crystal structure of the archaeal molybdate transporter ModB_2_C_2_ in complex with its SBP ModA from *A. fulgidus* [33]. Specificity is evident even in zinc ABC transporters expressed within the same organism. However, there is some evidence that such systems are not purely redundant. For example, while the zinc transporters are redundant for *Listeria monocytogenes* grown in media, they appear to play distinct roles in virulence [22]. This suggests that the appearance of redundancy may depend on the conditions under which it is evaluated.

While the AztC and ZnuA flexible loops are not essential for transport, there was a slight growth defect in the Δ*znuA/*Δ*loop aztC* mutant. It was observed previously that the flexible loop of AztC was required for the transfer of zinc from the metallochaperone AztD to AztC [28]. The growth lag could be caused by the inability of AztC to acquire zinc from AztD, which appears to act as a reservoir of zinc in the periplasm. Transfer of zinc from AztD may be faster or more efficient than its acquisition from solution by AztC. This is consistent with the observation of a similar growth defect in the Δ*aztD/*Δ*znuA* strain. In the case of *Salmonella enterica* ZnuA and its chaperone ZinT, a growth defect of a loop-deleted ZnuA mutant was only evident in the background of the null *zinT* mutation [32]. Thus, in both *P. denitrificans* and *S. enterica* it seems that the flexible loop is important for interaction of the SBP with its metallochaperone, even though ZinT and AztD share no structural similarities.

In the course of analyzing the sequence and structural features of cluster A-I SBPs, a clear outlier was detected in ZrgA from *V. cholerae*. This organism also encodes a typical *znuABC* operon, which appears to play the predominant role in zinc acquisition from media, although both operons function in this capacity. Furthermore, both appear to be equally important in contributing to virulence [23]. A ZrgA homologue from *P. aeruginosa* was later identified through a transcriptional analysis of a Δ*znuA* mutant [34]. Like *V. cholerae* ZrgA, it was found to be transcriptionally regulated by Zur in a zinc-dependent manner. Although its function in zinc acquisition was not directly evaluated, the Δ*znuA* mutant of *P. aeruginosa* exhibited a surprisingly mild growth defect in zinc-limited media, suggesting that another system may participate in this function. While much remains to be determined regarding their importance and specificity in zinc acquisition across multiple species, the early data suggests that ZrgA-like proteins represent a new family of metal binding proteins associated with ABC transporters. It will be of particular interest to confirm this by structural, functional and mechanistic studies. Furthermore, the role of ZrgA in *V. cholerae* virulence and its conservation in other human pathogens brings up the exciting possibility that it may represent a new target for the development of novel antimicrobials.

## 4. Materials and Methods

### 4.1. Strains and Media

See Supplementary Tables S2 and S3 for the list of strains and primers used, respectively. *E. coli* was grown at 37 °C in Luria-Bertani (LB) and *P. denitrificans* at 30 °C in minimal medium. The minimal medium was made by combining the following components at the specified concentration and adjusting the pH to 7.4: 1X M9 medium, 10X trace elements solution, 0.4% glucose, 1 mM MgSO_4_, 0.3 mM CaCl_2_ and 0, 10 or 50 µM ZnSO_4_. The 10X M9 medium stock was prepared by dissolving in Milli-Q water: 0.42 M Na_2_HPO_4_, 0.22 M KH_2_PO_4_ and 85.5 mM NaCl, in a glass bottle previously washed with 1 M nitric acid. This medium was sterilized in the autoclave. For the 100× trace elements solution stock: 17.1 mM EDTA, 3.07 mM FeCl_3_·6H_2_O, 76.3 µM CuCl_2_·2H_2_O, 42 µM CoCl_2_·6H_2_O, 162 µM H_3_BO_3_, 8.1 µM MnCl_2_·6H_2_O were dissolved in water. The resulting solution was vacuum filter sterilized. Additionally, stock solutions of 100 mM ZnSO_4_, 1 M MgSO_4_, 1 M CaCl_2_ were independently prepared and filter sterilized.

### 4.2. Construction of P. denitrificans Deletion Mutant Strains

Scarless deletions in the genome of the D-loop and Z-loop regions from *aztC*, D-loop from *znuA* and deletions of *aztAB* and *znuBC* genes were done by the PCR amplification of 600–700 bp flanking sequences of the target genes/regions and assembling into the *Eco*RI digested suicide vector pk18mobsacB (Km^r^) [35] using the Gibson cloning method [36]. Then, pk18mobsacB constructs were transformed into *E. coli* S17-1. Triparental mating was done to transfer the plasmid constructs from *E. coli* S17-1 to *P. denitrificans* WT, Δ*aztC*, Δ*znuA*, or Δ*aztC/*Δ*znuA* strains with the assistance of the conjugative plasmid pk2013 (Km^r^). Cells were streaked in high salt agar plates and then grown in low salt media. Since pk18mobsacB contains the counter-selectable sucrose sensitivity marker sacB, *P. denitrificans* double recombinants were selected by streaking cells in 6% sucrose LB agar. Verification of double crossover recombinants after sucrose selection was done by streaking the same colony in replica plates, first passing each colony in LB-rifampicin kanamycin and then in LB-rifampicin agar plates. All plasmids and *P. denitrificans* mutants constructed were confirmed by DNA sequencing.

### 4.3. Reverse Transcriptase Polymerase Chain Reaction (RT-PCR)

Cells were grown as above in zinc-limited media to mid-exponential phase (OD_600_~0.5). RNA was extracted and cDNA was synthesized from 5 µg of pure RNA in a 20 μL reaction volume using an iScript™cDNA synthesis kit (Bio-Rad^®^, Hercules, CA, USA). cDNA was used for PCR reactions. The primers (Supplementary Table S3) were designed to amplify the full-length target genes and were used at a final concentration 0.4 μM. PCR products were visualized on 1% agarose with ethidium bromide.

### 4.4. Cell Growth Determination

Overnight cultures of *P. denitrificans* WT and mutant cells from frozen stocks were grown in minimal media containing 10 µM ZnSO_4_. The pellet collected from 500 µL of overnight cultures was washed twice and resuspended in zinc depleted media. In a 96-well microplate, each sample from the previous resuspensions was inoculated in triplicate to an OD_600_ of 0.02 in 200 µL of minimal media with no zinc added or 50 µM ZnSO_4_. Cell growth at 30 °C was monitored for 24 h with an orbital shaking of 225 rpm in the EPOCH microplate reader (BioTek^®^, Winooski, VT, USA). A second passage in each condition was performed from washed cell resuspensions. Overnight cultures of each sample were inoculated to an OD_600_ of 0.02 in minimal media with no zinc added or 50 µM ZnSO_4_. Washing and growth curve procedure was repeated as described. The results from the second passage are presented in Figure 2, Figure 3 and Figure 4.

### 4.5. Colony Forming Units Assay

For CFU/mL quantification, one replicate of each sample from the 96 well-plate growth curves was used to make dilutions for plating. Cultures that reached saturation were diluted to 10^8^-fold or 10^6^ and the non-saturated to 10^5^-fold; 20 µL of each sample were spread uniformly in LB agar (1.5% agar) in 150 mm × 15 mm petri dishes with a glass spreader. Plates were incubated for 48 h at 30 °C. Pictures of the plates were taken with direct light on 60-2105 Gel Imaging System (Fotodyne Inc., Harland, WI, USA) and colonies were quantified with the Fiji software, using the cell counter plugin [37].

### 4.6. Sequence Alignments

Protein sequence alignments of characterized cluster A-1 solute binding proteins (Supplementary Table S1) were performed in Clustal Omega [38] (Supplementary Figure S2).

### 4.7. Phylogenetic Analysis of ZrgA

The protein sequence of ZrgA from *V. cholerae* (UniProtKB accession number Q9KP27) was used to perform a BLAST search of the UniProtKB database [39]. Sequences were filtered to include only those with E values below 10^−20^. These sequences were submitted to the Enzyme Function Initiative—Enzyme Similarity Tool [40,41,42] to generate a sequence similarity network, which was further processed and visualized in Cytoscape v3.8.1 [43]. Genome neighborhood networks were generated by Enzyme Function Initiative—Genome Neighborhood Tool.

## Supplementary Materials

Supplementary Materials can be found at https://www.mdpi.com/1422-0067/21/23/9098/s1.

## Abbreviations

SBP	Substrate binding proteins
ABC	ATP binding cassette
CFU	Colony Forming Units
