# Supplementary material for: Specificity of Interactions between Components of Two Zinc ABC Transporters in Paracoccus denitrificans
Source: Int J Mol Sci. 2020 Nov 30;21(23):9098. doi: 10.3390/ijms21239098 (PMC7731109; doi:10.3390/ijms21239098)
Supplement: Supplementary file 1 [file ijms-21-09098-s001.pdf]

Supplementary Information

Supplementary Table S1. Properties of the characterized cluster A-I solute binding proteins.

Protein	Organism	Ligand	PDB codes	UniProtKB code	References	KO	Sites	Kd	Binding site 1st coordination sphere	Flexible loop	Gram
ZnuA	*Acinetobacter baumannii*	Zn^{2+}	Not found	B0VNJ6	[1,2]	Zn[2]	nd	nd	nd	nd	-
ZnuA (YcdH)	*Bacillus subtilis*	Mn^{+2}	2O1E	O34966	Unpublished [3]	Zn[4]	nd	nd	His_2(His51 and His128)/ Glu_1 (Glu267)	Length: 16 a.a 5 histidines	+
ZnuA	*Brucella abortus* biovar 1	Zn^{2+}	Not found	Q576K1	[5]	Zn[5]	nd	nd	nd	nd	-
ZnuA2	*Candidatus Liberibacter asiaticus* str. psy62	Mn^{+2}<br>Mn^{+2} (mut)<br>Cd^{+2}<br>Zn^{+2}<br>Apo<br>Apo (mut)	4UDO, 4CL2<br>5ZHA, 5Z2K<br>6IXI<br>5AFS<br>4UDN<br>5Z35, 5Z2J	C6XF58	[6-9]	nd	1	Zn:430uM Mn:370uM [10]	His_2(His39, His106)<br>Asp_1(Asp247)<br>Glu_1(172)<br>H-bond: asp and arginine	No loop.	-
ZnuA	*Campylobacter jejuni*	Zn^{2+}	Not found	Q0PBZ4	[11]	Zn[11]	nd	nd	nd	Length: 14 a.a. 8 histidines	-
YtgA	*Chlamydia trachomatis*	Fe^{+3}	6NSI	Q9S529	[12]	nd	1	nd	His_3(His75, His141, His207)<br>Asp_1(299)	No major loop structure<br><br>The loop connecting alpha 9 and B6 on the surface of the C-terminal domain dwells directly above the Fe(III).	-
ZnuA	*Escherichia coli*	Zn^{+2}<br><br>Co^{+2}<br>Apo	2OSV, 2OGW, 2PS0<br>2PRS<br>2PS9<br>2PS3	P39172	[13] [14] [15]	Zn[16]	2	Zn: < 20 nM [15]	His_3 (His78, His161, His225)<br>Glu_1(Glu77).	Length: 21a.a 6 histidines	-
ZnuA (PZP1)	*Haemophilus influenzae* Rd KW20 (serotype d)	Zn^{2+}	Not found	P44526	[17]	Zn[17]	nd	nd	nd	Length: 50 a.a. 25 hisitidines[18]	-
Lmo1671	*Listeria monocytogenes* EGD-e	unknown	Not found	Q8Y6L3	none	nd	nd	nd	nd	Loop with histidines[19]	+
MntA	*Listeria monocytogenes* EGD-e	Cd^{+2}<br>Mn^{+2}<br>Apo	5JPD<br>5I4K<br>5HX7	Q8Y653	Unpublished[20]	nd	nd	nd	His_2 (His67, His140)<br>Glu_1(206)<br>Asp_1 (281)	No loop	+
ZinA	*Listeria monocytogenes*	Zn^{2+}	Not found	Q8YAH3	[21]	Zn[21]	nd	nd	nd	nd	+
MntC	*Neisseria gonorrhoeae*	Zn^{2+}<br>Mn^{2+}	Not found	B4RIV9	[22]	Zn, Mn[22]	1	Zn^{2+}: 104 ±5 nM<br>Mn^{2+}: 100±8 nM[22]	nd	Histidine rich loop[22]	-
AztC	*Paracoccus denitrificans*	Zn^{+2}<br>Zn^{+2} (mut)<br>Apo	5W57<br>5KZJ<br>5W56	A1B2F3	[23]	Zn[24]	1	Zn: 0.3 ± 0.1 nM[24]	His_3(His61, His138, His204)<br>Asp_1(279)	Length: 15 a.a 3 histidines	-
ZnuA	*Paracoccus denitrificans*	Zn^{2+}	Not found	A1B9L0	[24]	Zn[24]	5	Main binding site Zn: 1nM [24]	nd	Length: 38 a.a. 15 Histidines	-
ZnuA	*P. aeuroginosa* PAO1: PA5498	Zn^{2+}	Not found	Q9HT75	[18]	nd	2	Zn: 22.6±6.4 nM[18]	His_3(His60, His140 and His204)	Length: 15 a.a. 5 Histidines	-

									Water molecule<br>Based on<br>homology[18]		
ZnuA	*Salmonella enterica* subsp. *enterica* serovar Typhimurium	Zn^{+2}<br>Zn^{+2}/PH<br>Apo<br>(mut)	2XQV,<br>2XY4<br>4BBP<br>2XH8	Q8ZNV8	[25,26]	Zn[27]	2	1st site:<br><20nM<br>2nd site: uM range[28]	His3(His140, His147, His211)<br>Glu_1(Glu59)	Length: 21a.a<br>3 histidines	-
SitA	*Salmonella enterica* subsp. *enterica* serovar Typhimurium str. LT2	Apo	5JG7	Q7CPX8	Unpublished[29]	nd	nd	nd	nd	Length: 11 a.a<br>No histidines	-
MntC	*Staphylococcus aureus*	Fe^{+3}/co structure with mAB 305-78-7<br>Zn^{+2}<br>Mn^{+2}<br>Apo/antagonistic fab fragment	5HDQ<br><br>4NNO<br>4K3V<br>4NNP	A0A0H3JTS7	[30-32]	Mn[33]	1	Mn^{+2}: ~ 4 nM[32]	Mn^{+2}/Fe^{+3}<br>His2(His50, His123)<br>Asp_1(Asp264)<br>Glu_1(Glu189)<br>**Zn^{+2}:**<br>His2(His67, His140)<br>Asp_1(Asp281)<br>Glu_1(Glu206)	No major loop structure<br><br>Peptides 28-51 and 37-51 include a flexible loop with one of the four Mn+2 coordinating residues :H50<br><br>residues covered by peptides 111-125 and 111-126. They include another flexible loop and another Mn2+ -coordinating residue, H123	+
SitA	*Staphylococcus pseudintermedius*	Zn^{+2}<br>Mn^{+2}	4OXQ<br>4OXR	NCBI WP_014614644.1	[34]	nd	1	Mn^{+2} and Zn^{+2}: Low nM range.[34]	His_2(His64, His137)<br>Glu_1(Glu203)<br>Asp_1(Asp278)	No Loop	+
SitA	*Sinorhizobium meliloti*	Mn^{2+}<br>Fe^{2+}	Not found	F7XAF4	[35]	Mn, Fe(II)[35]	nd	nd	nd	nd	-
Lmb	*Streptococcus agalactiae* NGBS572	Zn^{+2}<br>Zn^{+2}<br>(mut)	3HJT<br>4H0F	Q9ZHG8	[36],[37]	Zn[38]	nd	nd	His_3(His66, His142 and His206).<br>Glu_1(Glu281).	Length: 12a.a<br>No histidines<br>Residue range: 124-135	+
AdcA	*Streptococcus agalactiae* A909 serotype Ia	Zn^{2+}	Not found	Q8E128	[38]	Zn[38]	nd	nd	3 Histidines<br>1 Glutamate<br>Predicted from sequence homology. [39]	Length: 11 a.a.<br>6 Histidines	+
PsaA	*Streptococcus pneumoniae*	Apo<br>Mn^{+2}<br>Zn^{+2}<br>Cd^{+2}	3ZK8<br>3ZK7<br>3ZK9<br>3ZKA<br>3ZTT<br>1PSZ<br>4UTO<br>4UTP	P0A4G2	[39-41]	Mn[42]	nd	231 nM Zn, 3.3 nM Mn[43]	His_2(His67, His139)<br>Glu205<br>Asp280	7 a.a<br>No histidines<br>Residue range: 129-135	+
AdcA	*Streptococcus pneumoniae*	Zn^{2+}	Not found	Q8CWN2	[44,45]	Zn[45]	2	Zn^{+2}: High Affinity site: 4±0.1 nM<br>Low Affinity Site: 228±88 nM[46]	His_3(His63, His140, His204)<br>Glu_1(Glu279)<br>Based on homology.[46]	Histidine Rich Loop is present.	+
AdcAII (Lmb)	*Streptococcus pneumoniae*	Zn^{+2}	3CX3	Q8DQ09	[19]	Zn [45]	nd	nd	His_3(His71, His147, His211)<br>Glu_1(Glu286)	Length: 13 a.a<br>No histidines<br>Residue range: 129-142	+
MtsA	*Streptococcus pyogenes*	Fe^{+3}	3HH8	P0A4G4	[46]	Mn, Fe[47,48]	1	4.3 µM Fe(II)	His_2(His68, His140)	No loop	+

								50 μM Mn	Glu_1(Glu206) Asp_1(Asp281)		
Lmb (Lbp)	*Streptococcus pyogenes* Serotype M1	Zn^{+2}	3GI1	Q99XV3	[49]	Zn[50],[51]	1	nd	His_3(His66, His142, His206) Glu_1(Glu281)	Length: 15 a.a. No histidine Residue range: 123-137	+
TroA	*Streptococcus suis*	Zn^{+2}	3MFQ	A4VY63	[52]	Mn[53]	1	434nM Zn 254nM Mn[53]	His_3(His76, His139, His205) Asp_1(Asp289)	Length: 4a.a Residue range: 126-129	+
ZnuA	*Synechocystis* sp. PCC 6803 substr. Kazusa	Zn^{+2} Zn^{+2} (mut) Apo (mut)	1PQ4 2OV3 2OV1	P73085	[54,55]	nd	4	7 nM, 9 μM Zn[55]	His_3(His83, His179, His243) Water molecule	Length: 34 a.a 8 histidines	-
MntC	*Synechocystis* sp. PCC 6803	Mn^{+2} Mn^{+2} (mut) Zn^{+2}, Mn^{+2}	1XVL 4IRM 3UJP	Q79EF9	[56,57]	Mn[58]	nd	nd	His_2(His89, His154) Glu_1(Glu220) Asp_1(Asp295)	No loop	-
TroA	*Treponema pallidum*	Zn^{+2} Apo	1TOA 1K0F	P96116	[59,60]	nd	1	23nM Zn 7.1nM Mn[61],[62]	His_3(His68, His133, His 199) Asp_1(Asp279)	4a.a No histidines	-
VC_2081 (ZnuA)	*Vibrio cholerae*	Zn^{2+}	Not found	Q9KQB9	[63]	Zn[63]	nd	nd	nd	nd	-
VC_2552 (ZrgA)	*Vibrio cholerae*	Zn^{2+}	Not found	Q9KP27	[63]	Zn[63]	nd	nd	nd	nd	-
YfeA	*Yersinia pestis*	Zn^{+2} Fe^{+3} Mn^{+2} Apo	5UXS 5UXU 5UY0 6Q1D 5UYG 5UYD 5UYA 5UYW 5UYV 5UY4 5UYE 5UYB 5UY5 5UYH 5UYF 5UYC 6Q1C	Q56952	[64-65]	Fe[66]	2	17.8 nM Mn, 6.6 nM Zn[67] Iron nd	His_2(His76, His141) Glu_1(207(holo), Glu256(apo)) Asp_1(Asp282)	Length: 21 a.a. Residue range: 119-140	-
ZnuA	*Yesinia pestis*	Zn^{2+}	Not found	Q8ZEU2	[67,68]	Zn[67]	nd	nd	His_3(His62, His153, His217) Water molecule Based on homology modeling.[69]	Histidine Rich loop present.	-

Supplementary Table S2. Strains used in this study.

Strain/plasmid	Genotype/ description	Source
PD1222	*P. denitrificans* Wild-type, Rm^r	A gift from Dr. Stephen Sprio UT Dallas
	PD1222 mutant, Δ*aztC*, Rif^r	[24]
	PD1222 mutant, Δ*znuA*, Rif^r	[24]
	PD1222 mutant, Δ*aztC*, Δ*znuA* Rif^r	[24]
	PD1222 mutant, ΔD-loop *aztC*, Δ*znuA*, Rif^r	This work
	PD1222 mutant, ΔZ-loop *aztC*, Δ*znuA*, Rif^r	This work
	PD1222 mutant, ΔD-loop *znuA*, Δ*aztC*, Rif^r	This work
	PD1222 mutant, Δ*aztC* Δ*znuBC*, Rif^r	This work
	PD1222 mutant, Δ*znuA* Δ*aztBA*, Rif^r	This work
S17-1	*E. coli,* with derivative RP4 plasmid integrated, for incorporation of plasmid constructs into *P. denitrificans.*	A gift from Dr. Stephen Sprio UT Dallas
PRK2013	*E. coli* with pRK2013 plasmid, which contains transfer system RK2 for mobilization of non-self-transmissible plasmids	A gift from Dr. Stephen Sprio UT Dallas
Plasmids		
pK18mobsacB	Gene replacement vector, sacB, Km^r	
	pK18mobsacB, with upstream and downstream 600bp flanking sequences of *D-loop* from *aztC*, Km^r	This work
	pK18mobsacB, with upstream and downstream 600bp flanking sequences of *Z-loop* from *aztC*, Km^r	This work
	pK18mobsacB, with upstream and downstream 600bp flanking sequences of *loop* from *znuA*, Km^r	This work
	pK18mobsacB, with upstream and downstream 600bp flanking sequences of *znuBC*, Km^r	This work
	pK18mobsacB, with upstream and downstream 600bp flanking sequences of *aztBA*, Km^r	This work

Supplementary Table S3. Primers used in this study.

Primer	Primer sequence 5′ to 3′
UP600aztAB_FWD	gaaacagctatgacatgattacgaacctcgggtcggatccgg
UP600aztAB_REV	cgatgcggaagagccagtctttcatcactggatagcggactc
DWN600aztAB_FWD	caaaatatcgagtccgctatccagtgatgaaagactggctcttc
DWN600aztAB_REV	gaggatccccgggtaccgagctcgaacacggtgcggcggtcttg
UP600znuBC_FWD	cagctatgacatgattacggggttatatcaccatccagacgctgc
UP600znuBC _REV	caccgaggccggcctgtgacctacgtatcggagcgggcggaaa
DWN600znuBC_FWD	tttccgcccgctccgatacgtaggtcacaggccggcctcggtg
DWN600znuBC _REV	atccccgggtaccgagctcgacgacgggaatatcggtcacc
UP600Loop_znuA_FWD	ctatgacatgattacggcccatggcgcgatgcgatt
UP600Loop_znuA_REV	catgaggatcggtgccggcatagtcccggcgatgcg
DWN600Loop_znuA_FWD	catcgccgggactatgccggcaccgatcctcatgcct
DWN600Loop_znuA_REV	ccgggtaccgagctcgtgaaagcgaaaaggcgggc
UP600D-Loop_aztC_FWD	gctatgacatgattacgcggcgcggttctgggtgaacg
UP600D-Loop_aztC_REV	tggggatcatgcgcgccgccgccgcccggctcc
DWN600D-Loop_aztC_FWD	gagccgggcggcggcggcgcgcatgatccccatgcc
DWN600D-Loop_aztC_REV	ccgggtaccgagctcggtctccttgatggggttcggggg
UP600Z-Loop_aztC_FWD	ctatgacatgattacgcaggcggaaccgctggacg
UP600 Z-Loop_aztC_REV	cacatcggccgcagcggcctgcggcgacaggaaatgcac
DWN600Z-Loop_aztC_FWD	tttcctgtcgccgcaggccgctgcggccgatgtg
DWN600Z-Loop_aztC_REV	cgggtaccgagctcgacgagatgccgctgcggatgaact
znuA_RT-PCR_FWD	actatcatatgatccgaccttcttccctt
znuA_ RT-PCR _REV	actatggtaccctagtcagccccaagg
znuB_ RT-PCR _FWD	ttatttcctgcaaaccact
znuB_ RT-PCR _REV	atgcttgacgatttcctga
znuC_ RT-PCR _FWD	tcaatgcggatgggcgca
znuC_ RT-PCR _REV	gtgacgccgctgatttgc
aztA_ RT-PCR _FWD	atgcccagaaccgatccga
aztA_ RT-PCR _REV	tcacgccacccctctgac
aztB_ RT-PCR _FWD	atggtcgacggggtcgt
aztB_ RT-PCR _REV	tcaggccgtccgatgat
aztC_ RT-PCR _FWD	atgaaagactggctcttccgc
aztC_ RT-PCR _REV	gaaatctggtcagcgggc

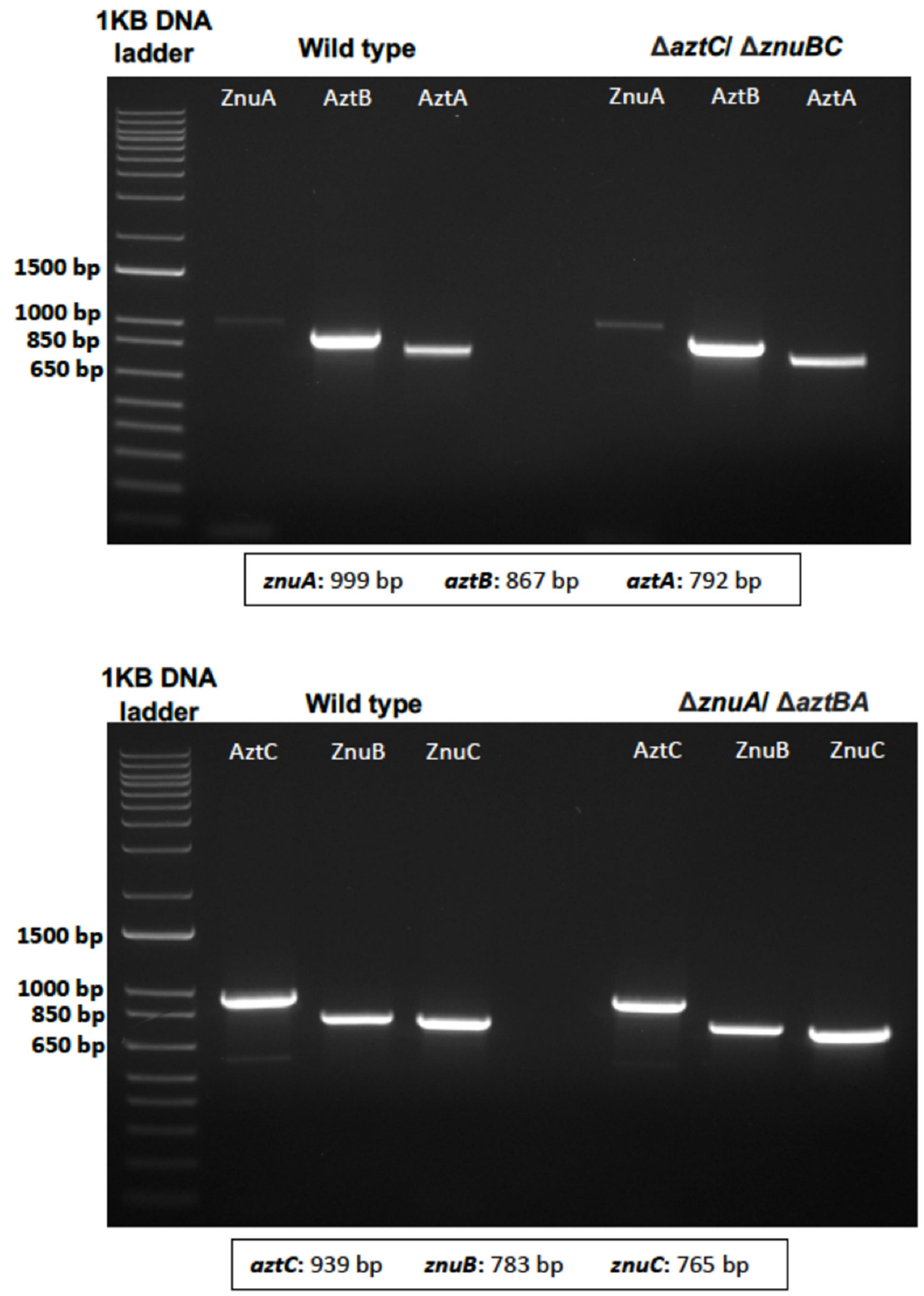

Supplementary Figure S1. RT-PCR of full-length ABC transporter genes from WT and hybrid transporter strains.

```
                                    1        10        20        30        40        50
V.cholerae_VC2552/25-242            SDHQHRQHEAHVHGQVELNIAQ..DGHDLLLEITAPGADVVGFEHAPQDDA..............QK....QA
C.jejuni_ZnuA/29-296                TSSNLVSVSIAPQAFFIKKIAANTLDVNVIL...PPNSNEHNFEFKPSTMKKLEKSDIYFTIGLEFE...KVF
Synechocystis_ZnuA/46-334           ADAMDITVSIPPQQYFLEKIGGDLVRVSVLV...PGNNDPHTYEPKPQQLAALSEAEAYVLIGLGFE...QPW
L.monocytogenes_Lmo1671/33-315      SDQLTVYTTVYPLQYLTEQIGGKYVDVHSIY...PPGSDAHSFEPTQKDMMKIADSDLFFYIGLGME....GF
S.agalactiae_AdcA/29-311            DSKLKVVTTFYPVYEFTKNVVGDKADVSMLI...KAGTEPHDFEPSTKNIAAIQDSNAFVYMDDNME....TW
S.pneumoniae_AdcA/26-307            DGKLNIVTTFYPVYEFTKQVAGDTANVELLI...GAGTEPHEYEPSAKAVAKIQDADTFVYENENME....TW
B.subtilis_ZnuA/34-315              GDKLHVVTTFYPMYEFTKQIVKDKGDVDLLI...PSSVEPHDWEPTPKDIANIQDADLFVYNSEYME....TW
L.monocytogenes_ZnuA/32-312         KDKLKVVTTFYPMYDFTKNVAGDNASIEMLI...DAGTEPHDYEPSAKDIAKIEAADVFVYNSEDME....TW
S.pneumoniae_AcdAII/35-310          GKGMKIVTSFYPIYAMVKEVSGDLNDVRM.I...QSSSGIHSFEPSANDIAAIYDADVFVYHSHTLE....SW
S.agalactiae_Lmb/30-305             TQGMSVVTSFYPMYAMTKEVSGDLNDVRM.I...QSGAGIHSFEPSVNDVAAIYDADLFVYHSHTLE....AW
S.pyogenes_Lmb/30-305               TQGMSVVTSFYPMYAMTKEVSGDLNDVRM.I...QSGAGIHSFEPSVNDVAAIYDADLFVYHSHTLE....AW
S.aureus_MntC/30-308                NGKLKVVTTNSILYDMAKNVGGDNVDIHSIV...PVGQDPHEYEVKPKDIKKLTDADVILYNGLNLETG.NGW
L.monocytogenes_MntA/30-308         DGKLNVVATYSILADIVKNVGGNKIELHSIV...PVGVDPHEYDPLPANIQSAADADLIFYNGLNLETG.NGW
S.pyogenes_MtsA/31-308              SDKLKVVATNSIIADMTKAIAGDKIDLHSIV...PIGQDPHEYEPLPEDVEKTSNADVIFYNGINLEDGGQAW
S.pneumoniae_PsaA/30-307            GQKLKVVATNSIIADITKNIAGDKIDLHSIV...PIGQDPHEYEPLPEDVKKTSEADLIFYNGINLETGGNAW
Ca.L.asiaticus_ZnuA2/21-293         TQKKVVLSSFSIIGDITQNIAKDLVTVTTLV...EAGNDSHSYQVTSADAIKIQNADLILCNGLHLE....ET
Synechocystis_MntC/52-322           EEKKKVLTTFTVLADMVQNVAGDKLVVESIT...RIGAEIHGYEPTPSDIVKAQDADLILYNGMNLE....RW
Y.pestis_YfeA/39-309                AKKFKVVTTFTIIQDIAQNIAGDVAVVESIT...KPGAEIHDYQPTPRDIVKAQSADLILWNGMNLE....RW
S.enterica_SitA/27-297              KEKFKVITTFTVIADMAKNVAGDAAEVSSIT...KPGAEIHEYQPTPGDIKRAQGAQLILANGLNLE....RW
S.meliloti_SitA/29-299              AEKLKAVTTFTVIADMAQNVAGDAAVVESIT...KPGAEIHNYQPTPRDILKAHDADLILWNGLNLE....LW
Rhizobium/Agrobacterium_TroA/24-294 QEKPKVVTTFTIIADMARNVAGDAAEVESIT...KPGAEIHNYQPTPRDILKARKADLVLRNGLNLE....LW
N.gonorrhoeae_MntC/19-302           AAPLPVVTSFSILGDVAKQIGGERVAVQSLV...GANQDTHAYHMTSGDIKKIRSAKLVLLNGLGLE....AA
P.denitrificans_AztC/24-306         AEPLDVVATFSIIGDFAAKVGGDRIRLNVLV...GPDSDTHVYEPRPADAIALAGADVVLTNGLEFE....GF
Rhizobium/Agrobacterium_ZnuA/26-324 AAELKVVASFSIIADFAKNVGGNRVEITTIV...GPDGDAHVYEPRPADAVAVSKADVVLVNGLAFE....GF
C.trachomatis_YtgA/38-320           DESIYVLSMNRMICDCVSRITGDRVKNIVLI...DGAIDPHSYEMVKGDEDRMAMSQLIFCNGLGLEHS..AS
S.suis_TroA/40-316                  S.KPRVAVTTSFLNDMVYQLAGDEVERDLLI...PAGEDPHLYVAKSSDLSKLQKADLVLYHGLHFE....GK
T.pallidum_TroA/32-306              G.KPLVVTTIGMIADAVKNIAQGDVHLKGLM...GPGVDPHLYTATAGDVEWLGNADLILYNGLHLE....TK
A.baumannii_ZnuA/17-274             GWSQGLVVSTHPIYLIAKEITKGVEEPQLLL...Q.GQSGHDVQLTPAHRKAINDASLVIWLGKAHE....AP
P.denitrificans_ZnuA/22-323         AEVPRVVTDIPVVGAMVQQVMGDLGQPEILL...QAGGDPHSYQLRPSQARSLQDADLLIWVGPELT....PW
B.abortus_ZnuA/24-332               GEREGVVVSIKPLHSIVSAVMQGVGKPKLIV...QGAGSEHVYSLKPSDAEAIEHAKVIFWAGPSME....TF
P.aeruginosa_ZnuA/23-304            RAEVSVLTSIKPLQLVAAAIQDGVGQPDVLL...PPGASPHQYALRPSDVRRLREVQLFYWIGPDLE....NF
V.cholerae_VC2081/21-294            ASAIEVLTSIKPIQMITYELMLGTGTPDVLL...PSGASPHDYALRPSDVKRIQQADLVIWFGQDLE....PF
H.influenzae_ZnuA/20-335            MANADVLASVKPLGFIVSSIADGVTGTQVLV...PAGASPHDYNLKLSDIQKVKSADLVVWIGEDID....SF
Y.pestis_ZnuA/25-317                LASAAVVTSIRPLGFIAAAIADGVLPTEVLL...PDGASPHDYALRPSDVQRLRSAELVIWVGPEME....AF
E.coli_ZnuA/23-307                  AADAAVVASLKPVGFIASAIADGVTETEVLL...PDGASEHDYSLRPSDVKRLQNADLVVWVGPEME....AF
S.enterica_ZnuA/42-330              AADAAVVASLKPLGFIASAIADGVTDTQVLL...PDGASEHDYSLRPSDVKRLQGADLVVWVGPEME....AF
```

```
                                        60         70         80        90        100       110
V.cholerae_VC2552/25-242            LEKALETLHHPE....KLFALSDKAQCEKREVLIKHTLGGEEYQHSHAYGEDEEHEHQHGEGEHDHKA...HD
C.jejuni_ZnuA/29-296                TDKFKQNFS.KL....QVVNMQKNIALIQTH...........................DT...HEHSH..E..
Synechocystis_ZnuA/46-334           LEKLKAANA.NM....KLIDSAQGITPLEME............................K...HDHSHGEEEG
L.monocytogenes_Lmo1671/33-315      VDKAKKTLANEH......VTFVPTAEKLDLP...........................KDPD........AAE
S.agalactiae_AdcA/29-311            VPKVAKSVKSKK......VTTIKGTGDMLLT...........................KGVE........EEG
S.pneumoniae_AdcA/26-307            VPKLLDTLDKKK......VKTIKATGDMLLL...........................PGGE........EEE
B.subtilis_ZnuA/34-315              VPSAEKSMGQGH......AVFVNASKGIDLM...........................EGSE........EEH
L.monocytogenes_ZnuA/32-312         VPSVLKSLDSKK......LTVIDASKGIELV...........................EGTE........EED
S.pneumoniae_AcdAII/35-310          AGSLDPNLKKSK......VKVLEASEGMTLE...........................RVPG........LED
S.agalactiae_Lmb/30-305             ARDLDPNLKKSK......VDVFEASKPLTLD...........................RVKG........LED
S.pyogenes_Lmb/30-305               ARDLDPNLKKSK......VDVFEASKPLTLD...........................RVKG........LED
S.aureus_MntC/30-308                FEKALEQAGKSLKD.KKVIAVSKDVKPIYLN...........................GEEG...........
L.monocytogenes_MntA/30-308         FDRMLETADKSREDKNQVVELSKGVKPKYLT...........................EK.G...........
S.pyogenes_MtsA/31-308              FTKLVKNAQKTKN..KDYFAVSDGIDVIYLE...........................GA.S...........
S.pneumoniae_PsaA/30-307            FTKLVENAKKTEN..KDYFAVSDGVDVIYLE...........................GQ.N...........
Ca.L.asiaticus_ZnuA2/21-293         YMKYFTNLKKGT....KIITVTDGINPIGVS...........................EDTS...........
Synechocystis_MntC/52-322           FEQFLGNVK.DV....PSVVLTEGIEPIPIA...........................DGP............
Y.pestis_YfeA/39-309                FEKFFESIK.DV....PSAVVTAGITPLPIR...........................EGP............
S.enterica_SitA/27-297              FARFYQHLS.GV....PEVVVSTGVKPMGIT...........................EGP............
S.meliloti_SitA/29-299              FERFFQNFD.GV....PGVVVSEGVEPMGIA...........................EGP............
Rhizobium/Agrobacterium_TroA/24-294 FEKFLANLS.GV....PDVTVSDGIEPMAIN...........................GGA............
N.gonorrhoeae_MntC/19-302           DIQR.AVKQSKV....SYAEATKGIQPLKAE...........................E...........EG
P.denitrificans_AztC/24-306         LTRLIAASGTDA....AVATLTDGVETMEE..............................PGG..........
Rhizobium/Agrobacterium_ZnuA/26-324 LKRLIDTSGTKA....PVVELTKGVKPLRIS...........................DEPAGHAHPEAEEEA
C.trachomatis_YtgA/38-320           LRKH...LEGNP....KVVDLGQRL....LN...........................KN.............
S.suis_TroA/40-316                  MVEALEKTGV.........AVSKNFNAKDL............................................
T.pallidum_TroA/32-306              MGEVFSKLRGSR....LVVAVSETIPV...............................................
A.baumannii_ZnuA/17-274             LNKLLSNNK.......KAIALLDSGILSILP..................QRNT.....................
P.denitrificans_ZnuA/22-323         LERAATSLSAQS....EMLALLDLPATHRRD..................YAGG.......EHEHEHEH...EH
B.abortus_ZnuA/24-332               LDKPIDTLGEGA....KVVALGDAKGLTKLK..................FREGGPFE..AHDHGHGGSH...EE
P.aeruginosa_ZnuA/23-304            LPKVLAGRQGT......SVAVQDLPGMHLRK..................FVN...FEE.EEHAG..........
V.cholerae_VC2081/21-294            MSKLLEGRT.......SALTLSQVPNLA.....................LRE.......YG............
H.influenzae_ZnuA/20-335            LDKPISQIERK.....KVITIADLADVKPLL..................SKAHHEHFHEDGDHDHDHKH...EH
Y.pestis_ZnuA/25-317                LSKPLTQVAEN.....KQIALSQLPSVTPLL..................MKSDEHDEAEEGESGHH........
E.coli_ZnuA/23-307                  MQKPVSKLPGA.....KQVTIAQLEDVKPLL..................MKSIHGDDDDHDHA...........
S.enterica_ZnuA/42-330              MEKSVRNIPDN.....KQVTIAQLADVKPLL..................MKGADDDEDEHAHTGA.........
```

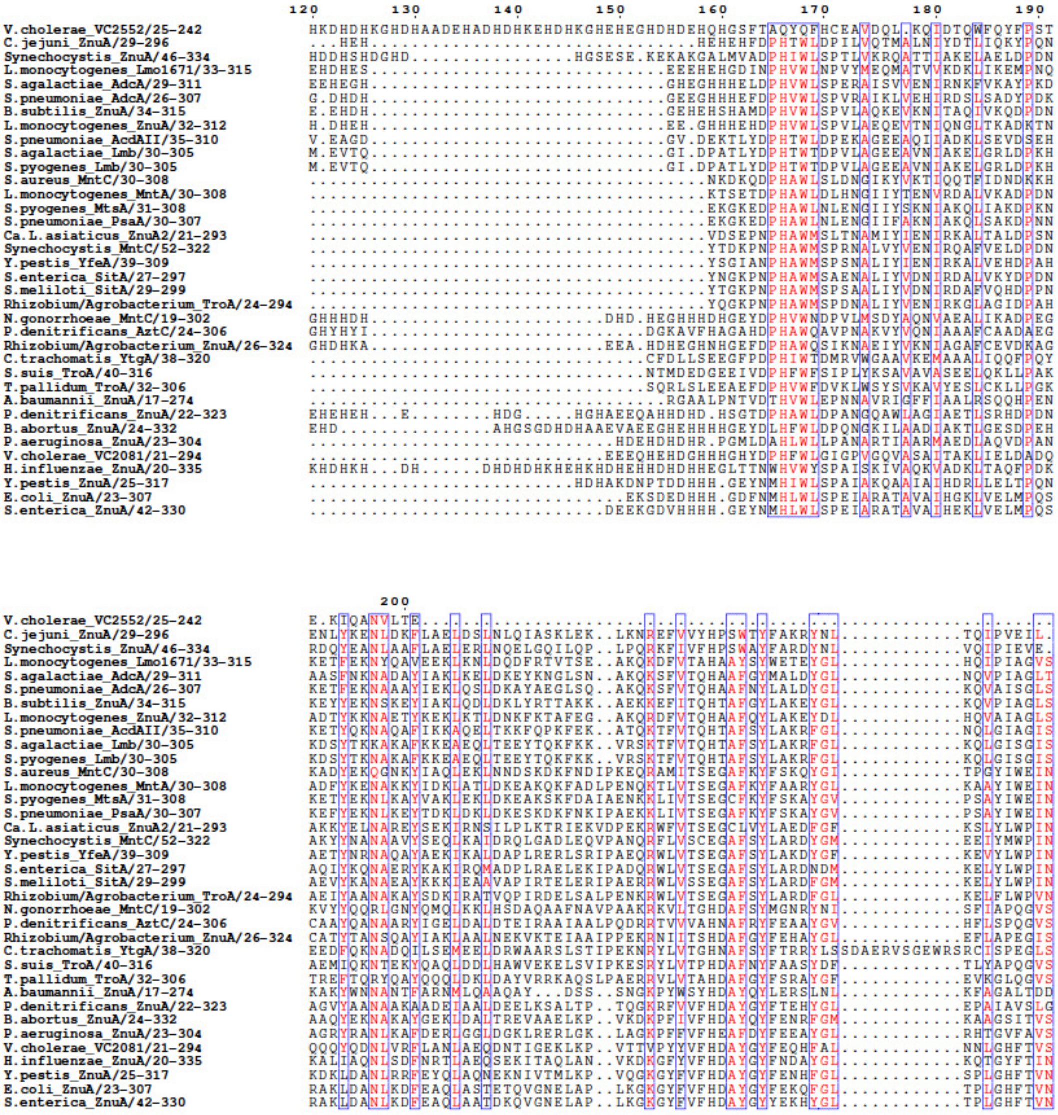

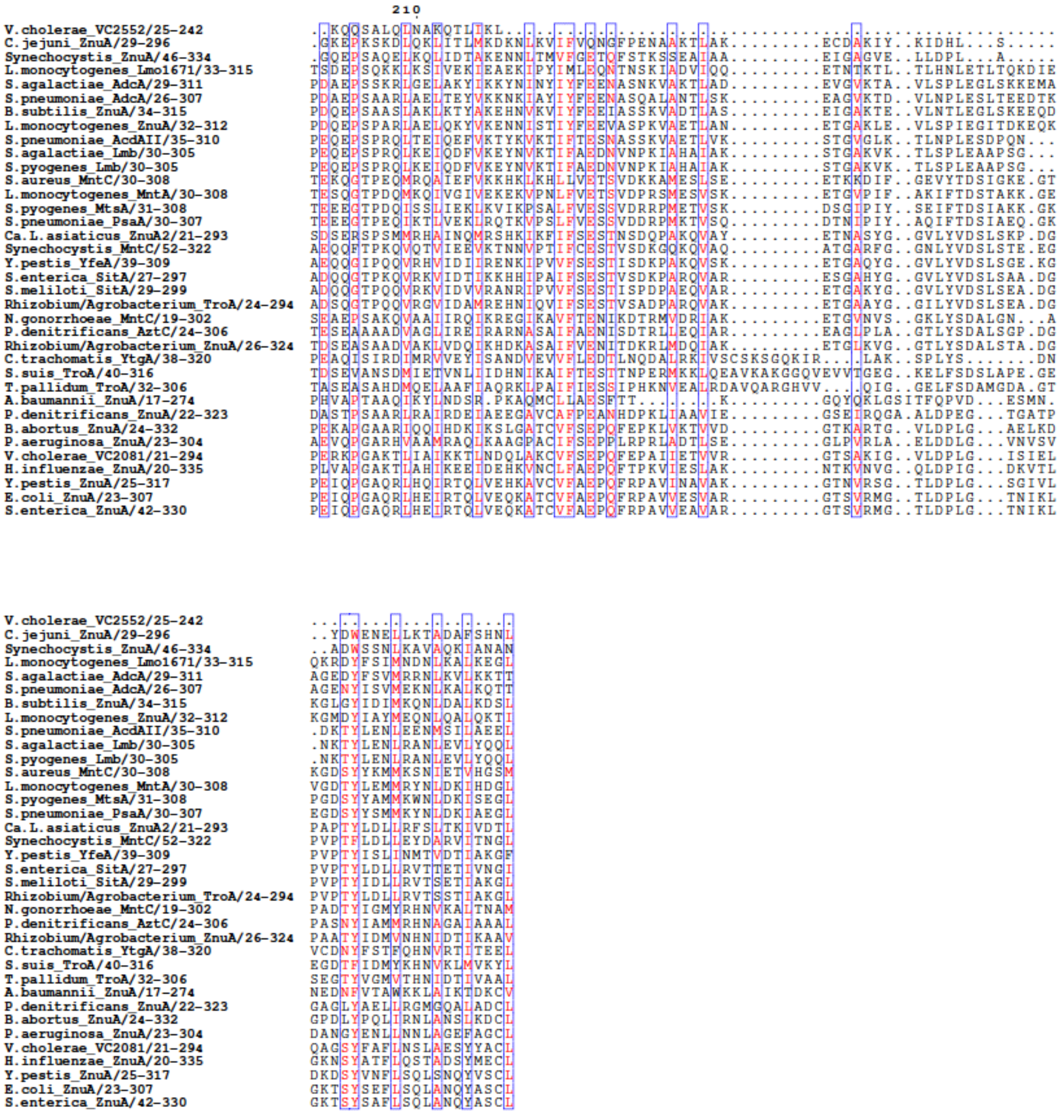

Supplementary Figure S2. Multiple sequence alignment of SBPs from Supplementary Table S1.

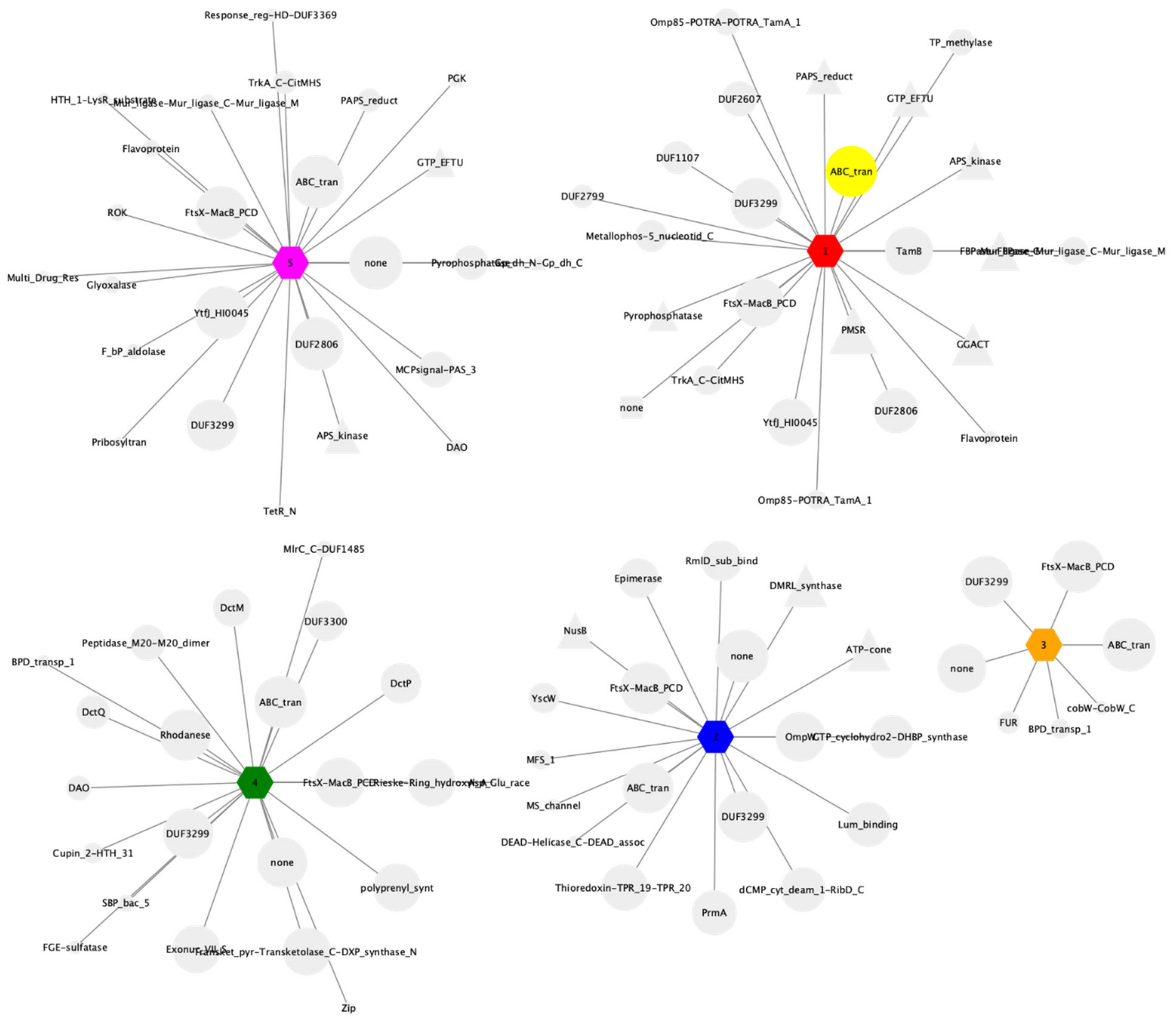

Supplementary Figure S3. Genome neighborhood network where the hub node represents each group indicated in Figure 5. The gray spoke nodes indicate the prevalence of Pfam [69] protein family genes within 10 genes of *zrgA* in at least 20% of genomes.

References:

1. Hood, M.I.; Mortensen, B.L.; Moore, J.L.; Zhang, Y.; Kehl-Fie, T.E.; Sugitani, N.; Chazin, W.J.; Caprioli, R.M.; Skaar, E.P. Identification of an *Acinetobacter baumannii* zinc acquisition system that facilitates resistance to calprotectin-mediated zinc sequestration. *PLOS Pathog* **2012**, *8*, e1003068, doi:10.1371/journal.ppat.1003068.
2. Hesse, L.E.; Lonergan, Z.R.; Beavers, W.N.; Skaar, E.P. The *Acinetobacter baumannii* Znu system overcomes host-Imposed nutrient zinc limitation. *Infect Immun* **2019**, *87*, e00746-00719, doi:10.1128/IAI.00746-19.
3. Forouhar, F.; Zhou, W.; Seetharaman, J.; Chen, C.X.; Fang, Y.; Cunningham, K.; Ma, L.C.; Janjua, H.; Xiao, R.; Baran M.C.; Liu, J.; Acton, T.B.; Montelione, G.T.; Junt, J.F.; Tong, L; Crystal structure of the metal-dependent lipoprotein YcdH from Bacillus subtilis, Northeast Structural Genomics Target SR583, unpublished, doi:10.2210/pdb2O1E/pdb.
4. Gaballa, A.; Wang, T.; Ye, R.W.; Helmann, J.D. Functional analysis of the *Bacillus subtilis* Zur regulon. *J Bacteriol* **2002**, *184*, 6508-6514, doi:10.1128/jb.184.23.6508-6514.2002.
5. Yang, X.; Becker, T.; Walters, N.; Pascual, D.W. Deletion of znuA virulence factor attenuates *Brucella abortus* and confers protection against wild-type challenge. *Infect Immun* **2006**, *74*, 3874-3879, doi:10.1128/IAI.01957-05.
6. Sharma, N.; Selvakumar, P.; Bhose, S.; Ghosh, D.K.; Kumar, P.; Sharma, A.K. Crystal structure of a periplasmic solute binding protein in metal-free, intermediate and metal-bound states from *Candidatus Liberibacter* asiaticus. *J Struct Biol* **2015**, *189*, 184-194, doi:10.1016/j.jsb.2015.01.012.
7. Sharma, N.; Selvakumar, P.; Saini, G.; Warghane, A.; Ghosh, D.K.; Sharma, A.K. Crystal structure analysis in Zn^{2+}-bound state and biophysical characterization of CLas-ZnuA2. *Biochim Biophys Acta* **2016**, *1864*, 1649-1657, doi:10.1016/j.bbapap.2016.08.016.
8. Saini, G.; Sharma, N.; Dalal, V.; Warghane, A.; Ghosh, D.K.; Kumar, P.; Sharma, A.K. The analysis of subtle internal communications through mutation studies in periplasmic metal uptake protein CLas-ZnuA2. *J Struct Biol* **2018**, *204*, 228-239, doi:10.1016/j.jsb.2018.08.013.
9. Kumar, P.; Dalal, V.; Sharma, N.; Kokane, S.; Ghosh, D.K.; Kumar, P.; Sharma, A.K. Characterization of the heavy metal binding properties of periplasmic metal uptake protein CLas-ZnuA2. *Metallomics* **2019**, *12*, 280-289, doi:10.1039/c9mt00200f.
10. Sharma, N.; Selvakumar, P.; Saini, G.; Warghane, A.; Ghosh, D.K.; Sharma, A.K. Crystal structure analysis in Zn^{2+}-bound state and biophysical characterization of CLas-ZnuA2. *Biochim Biophys Acta Proteins Proteom* **2016**, *1864*, 1649-1657, doi:org/10.1016/j.bbapap.2016.08.016.
11. Davis, L.M.; Kakuda, T.; DiRita, V.J. A *Campylobacter jejuni znuA* orthologue is essential for growth in low-zinc environments and chick colonization. *J Bacteriol* **2009**, *191*, 1631-1640, doi:10.1128/JB.01394-08.
12. Luo, Z.; Neville, S.L.; Campbell, R.; Morey, J.R.; Menon, S.; Thomas, M.; Eijkelkamp, B.A.; Ween, M.P.; Huston, W.M.; Kobe, B., et al. The structure and metal binding properties of *Chlamydia trachomatis* YtgA. *J Bacteriol* **2019**, *202*, e00580-19, doi:10.1128/jb.00580-19.

13. Li, H.; Jogl, G. Crystal structure of the zinc-binding transport protein ZnuA from *Escherichia coli* reveals an unexpected variation in metal coordination. *J Mol Biol* **2007**, *368*, 1358-1366, doi:10.1016/j.jmb.2007.02.107.
14. Chandra, B.R.; Yogavel, M.; Sharma, A. Structural analysis of ABC-family periplasmic zinc binding protein provides new insights into mechanism of ligand uptake and release. *J Mol Biol* **2007**, *367*, 970-982, doi:10.1016/j.jmb.2007.01.041.
15. Yatsunyk, L.A.; Easton, J.A.; Kim, L.R.; Sugarbaker, S.A.; Bennett, B.; Breece, R.M.; Vorontsov, II; Tierney, D.L.; Crowder, M.W.; Rosenzweig, A.C. Structure and metal binding properties of ZnuA, a periplasmic zinc transporter from *Escherichia coli*. *J Biol Inorg Chem* **2008**, *13*, 271-288, doi:10.1007/s00775-007-0320-0.
16. Graham, A.I.; Hunt, S.; Stokes, S.L.; Bramall, N.; Bunch, J.; Cox, A.G.; McLeod, C.W.; Poole, R.K. Severe zinc depletion of *Escherichia coli*: roles for high affinity zinc binding by ZinT, zinc transport and zinc-independent proteins. *J Biol Chem* **2009**, *284*, 18377-18389, doi:10.1074/jbc.M109.001503.
17. Lu, D.; Boyd, B.; Lingwood, C.A. Identification of the key protein for zinc uptake in *Hemophilus influenzae. J Biol Chem* **1997**, *272*, 29033-29038.
18. Pederick, V.G.; Eijkelkamp, B.A.; Begg, S.L.; Ween, M.P.; McAllister, L.J.; Paton, J.C.; McDevitt, C.A. ZnuA and zinc homeostasis in *Pseudomonas aeruginosa*. *Sci Rep* **2015**, *5*, 13139, doi:10.1038/srep13139.
19. Loisel, E.; Jacquamet, L.; Serre, L.; Bauvois, C.; Ferrer, J.L.; Vernet, T.; Di Guilmi, A.M.; Durmort, C. AdcAII, a new pneumococcal Zn-binding protein homologous with ABC transporters: biochemical and structural analysis. *J Mol Biol* **2008**, *381*, 594-606, doi:10.1016/j.jmb.2008.05.068.
20. Osipiuk, J.; Zhou, M.; Grimshaw, S.; Anderson, W.F. Metal ABC transporter from Listeria monocytogenes, unpublished, doi:10.2210/pdb5HX7/pdb.
21. Corbett, D.; Wang, J.; Schuler, S.; Lopez-Castejon, G.; Glenn, S.; Brough, D.; Andrew, P.W.; Cavet, J.S.; Roberts, I.S. Two zinc uptake systems contribute to the full virulence of *Listeria monocytogenes* during growth *in vitro* and *in vivo*. *Infect Immun* **2012**, *80*, 14-21, doi:10.1128/IAI.05904-11.
22. Lim, K.H.L.; Jones, C.E.; vanden Hoven, R.N.; Edwards, J.L.; Falsetta, M.L.; Apicella, M.A.; Jennings, M.P.; McEwan, A.G. Metal binding specificity of the MntABC permease of *Neisseria gonorrhoeae* and its influence on bacterial growth and interaction with cervical epithelial cells. *Infect Immun* **2008**, *76*, 3569-3576, doi:10.1128/IAI.01725-07.
23. Neupane, D.P.; Avalos, D.; Fullam, S.; Roychowdhury, H.; Yukl, E.T. Mechanisms of zinc binding to the solute-binding protein AztC and transfer from the metallochaperone AztD. *J Biol Chem* **2017**, *292*, 17496-17505, doi:10.1074/jbc.M117.804799.
24. Neupane, D.P.; Kumar, S.; Yukl, E.T. Two ABC transporters and a periplasmic metallochaperone participate in zinc acquisition in *Paracoccus denitrificans Biochem.* **2019**, *58*, 126-136, doi:10.1021/acs.biochem.8b00854.
25. Ilari, A.; Pescatori, L.; Di Santo, R.; Battistoni, A.; Ammendola, S.; Falconi, M.; Berlutti, F.; Valenti, P.; Chiancone, E. *Salmonella enterica* serovar Typhimurium growth is inhibited by the concomitant binding of Zn(II) and a pyrrolyl-hydroxamate to ZnuA, the

soluble component of the ZnuABC transporter. *Biochim Biophys Acta* **2016**, *1860*, 534-541, doi:10.1016/j.bbagen.2015.12.006.
26. Ilari, A.; Alaleona, F.; Petrarca, P.; Battistoni, A.; Chiancone, E. The X-ray structure of the zinc transporter ZnuA from *Salmonella enterica* discloses a unique triad of zinc-coordinating histidines. *J Mol Biol* **2011**, *409*, 630-641, doi:10.1016/j.jmb.2011.04.036.
27. Ammendola, S.; Pasquali, P.; Pistoia, C.; Petrucci, P.; Petrarca, P.; Rotilio, G.; Battistoni, A. High-affinity Zn^{2+} uptake system ZnuABC is required for bacterial zinc homeostasis in intracellular environments and contributes to the virulence of Salmonella enterica. *Infect Immun* **2007**, *75*, 5867-5876, doi:10.1128/iai.00559-07.
28. Ilari, A.; Alaleona, F.; Tria, G.; Petrarca, P.; Battistoni, A.; Zamparelli, C.; Verzili, D.; Falconi, M.; Chiancone, E. The Salmonella enterica ZinT structure, zinc affinity and interaction with the high-affinity uptake protein ZnuA provide insight into the management of periplasmic zinc. *Biochim Biophys Acta* **2014**, *1840*, 535-544, doi:10.1016/j.bbagen.2013.10.010.
29. Chang, C.; Zhou, M.; Shatsman, S.; Joachimiak, A.; Anderson, W.F. Crystal structure of putative periplasmic binding protein from Salmonella typhimurium LT2, unpublished, doi:10.2210/pdb5JG7/pdb.
30. Gribenko, A.V.; Parris, K.; Mosyak, L.; Li, S.; Handke, L.; Hawkins, J.C.; Severina, E.; Matsuka, Y.V.; Anderson, A.S. High resolution mapping of bactericidal monoclonal antibody binding epitopes on *Staphylococcus aureus* antigen MntC. *PLoS Pathog* **2016**, *12*, e1005908, doi:10.1371/journal.ppat.1005908.
31. Ahuja, S.; Rougé, L.; Swem, D.L.; Sudhamsu, J.; Wu, P.; Russell, S.J.; Alexander, M.K.; Tam, C.; Nishiyama, M.; Starovasnik, M.A., et al. Structural analysis of bacterial ABC transporter inhibition by an antibody fragment. *Struct.* **2015**, *23*, 713-723, doi:10.1016/j.str.2015.01.020.
32. Gribenko, A.; Mosyak, L.; Ghosh, S.; Parris, K.; Svenson, K.; Moran, J.; Chu, L.; Li, S.; Liu, T.; Woods, V.L., Jr., et al. Three-dimensional structure and biophysical characterization of *Staphylococcus aureus* cell surface antigen-manganese transporter MntC. *J Mol Biol* **2013**, *425*, 3429-3445, doi:10.1016/j.jmb.2013.06.033.
33. Horsburgh, M.J.; Wharton, S.J.; Cox, A.G.; Ingham, E.; Peacock, S.; Foster, S.J. MntR modulates expression of the PerR regulon and superoxide resistance in *Staphylococcus aureus* through control of manganese uptake. *Mol Microbiol* **2002**, *44*, 1269-1286, doi:10.1046/j.1365-2958.2002.02944.x.
34. Abate, F.; Malito, E.; Cozzi, R.; Lo Surdo, P.; Maione, D.; Bottomley, M.J. Apo, Zn^{2+}-bound and Mn^{2+}-bound structures reveal ligand-binding properties of SitA from the pathogen *Staphylococcus pseudintermedius Biosci Rep* **2014**, *34*, e00154, doi:10.1042/bsr20140088.
35. Chao, T.-C.; Becker, A.; Buhrmester, J.; Pühler, A.; Weidner, S. The *Sinorhizobium meliloti fur* gene regulates, with dependence on Mn(II), transcription of the *sitABCD* operon, encoding a metal-type transporter. *J Bacteriol* **2004**, *186*, 3609-3620, doi:10.1128/JB.186.11.3609-3620.2004.

36. Ragunathan, P.; Spellerberg, B.; Ponnuraj, K. Structure of laminin-binding adhesin (Lmb) from *Streptococcus agalactiae Acta Crystallogr D* **2009**, *65*, 1262-1269, doi:doi:10.1107/S0907444909038359.
37. Ragunathan, P.; Sridaran, D.; Weigel, A.; Shabayek, S.; Spellerberg, B.; Ponnuraj, K. Metal binding is critical for the folding and function of laminin binding protein, Lmb of *Streptococcus agalactiae*. *PLoS One* **2013**, *8*, e67517, doi:10.1371/journal.pone.0067517.
38. Moulin, P.; Patron, K.; Cano, C.; Zorgani, M.A.; Camiade, E.; Borezée-Durant, E.; Rosenau, A.; Mereghetti, L.; Hiron, A. The Adc/Lmb system mediates zinc acquisition in *Streptococcus agalactiae* and contributes to bacterial growth and survival. *J Bacteriol* **2016**, *198*, 3265-3277, doi:10.1128/JB.00614-16.
39. Couñago, R.M.; Ween, M.P.; Begg, S.L.; Bajaj, M.; Zuegg, J.; O'Mara, M.L.; Cooper, M.A.; McEwan, A.G.; Paton, J.C.; Kobe, B., et al. Imperfect coordination chemistry facilitates metal ion release in the Psa permease. *Nat Chem Biol* **2014**, *10*, 35-41, doi:10.1038/nchembio.1382.
40. Lawrence, M.C.; Pilling, P.A.; Epa, V.C.; Berry, A.M.; Ogunniyi, A.D.; Paton, J.C. The crystal structure of pneumococcal surface antigen PsaA reveals a metal-binding site and a novel structure for a putative ABC-type binding protein. *Struct.* **1998**, *6*, 1553-1561, doi:10.1016/s0969-2126(98)00153-1.
41. Begg, S.L.; Eijkelkamp, B.A.; Luo, Z.; Couñago, R.M.; Morey, J.R.; Maher, M.J.; Ong, C.L.; McEwan, A.G.; Kobe, B.; O'Mara, M.L., et al. Dysregulation of transition metal ion homeostasis is the molecular basis for cadmium toxicity in *Streptococcus pneumoniae Nat Commun* **2015**, *6*, 6418, doi:10.1038/ncomms7418.
42. Dintilhac, A.; Alloing, G.; Granadel, C.; Claverys, J.-P. Competence and virulence of *Streptococcus pneumoniae*: Adc and PsaA mutants exhibit a requirement for Zn and Mn resulting from inactivation of putative ABC metal permeases. *Mol Microbiol* **1997**, *25*, 727-739, doi:10.1046/j.1365-2958.1997.5111879.x.
43. McDevitt, C.A.; Ogunniyi, A.D.; Valkov, E.; Lawrence, M.C.; Kobe, B.; McEwan, A.G.; Paton, J.C. A Molecular mechanism for bacterial susceptibility to zinc. *PLOS Pathog* **2011**, *7*, e1002357, doi:10.1371/journal.ppat.1002357.
44. Bayle, L.; Chimalapati, S.; Schoehn, G.; Brown, J.; Vernet, T.; Durmort, C. Zinc uptake by *Streptococcus pneumoniae* depends on both AdcA and AdcAII and is essential for normal bacterial morphology and virulence. *Mol Microbiol* **2011**, *82*, 904-916, doi:10.1111/j.1365-2958.2011.07862.x.
45. Plumptre, C.D.; Eijkelkamp, B.A.; Morey, J.R.; Behr, F.; Couñago, R.M.; Ogunniyi, A.D.; Kobe, B.; O'Mara, M.L.; Paton, J.C.; McDevitt, C.A. AdcA and AdcAII employ distinct zinc acquisition mechanisms and contribute additively to zinc homeostasis in *Streptococcus pneumoniae Mol Microbiol* **2014**, *91*, 834-851, doi:10.1111/mmi.12504.
46. Sun, X.; Baker, H.M.; Ge, R.; Sun, H.; He, Q.Y.; Baker, E.N. Crystal structure and metal binding properties of the lipoprotein MtsA, responsible for iron transport in *Streptococcus pyogenes*. *Biochem.* **2009**, *48*, 6184-6190, doi:10.1021/bi900552c.
47. Janulczyk, R.; Pallon, J.; Björck, L. Identification and characterization of a *Streptococcus pyogenes* ABC transporter with multiple specificity for metal cations. *Mol Microbiol* **1999**, *34*, 596-606, doi:10.1046/j.1365-2958.1999.01626.x.

48. Janulczyk, R.; Ricci, S.; Björck, L. MtsABC is important for manganese and iron transport, oxidative stress resistance, and virulence of *Streptococcus pyogenes*. *Infect Immun* **2003**, *71*, 2656-2664, doi:10.1128/IAI.71.5.2656-2664.2003.
49. Linke, C.; Caradoc-Davies, T.T.; Young, P.G.; Proft, T.; Baker, E.N. The laminin-binding protein Lbp from *Streptococcus pyogenes* is a zinc receptor. *J Bacteriol* **2009**, *191*, 5814-5823, doi:10.1128/jb.00485-09.
50. Tedde, V.; Rosini, R.; Galeotti, C.L. Zn^{2+} uptake in *Streptococcus pyogenes*: characterization of *adcA* and *lmb* null mutants. *PLOS ONE* **2016**, *11*, e0152835, doi:10.1371/journal.pone.0152835.
51. Weston, B.F.; Brenot, A.; Caparon, M.G. The metal homeostasis protein, Lsp, of *Streptococcus pyogenes* is necessary for acquisition of zinc and virulence. *Infect Immun* **2009**, *77*, 2840-2848, doi:10.1128/IAI.01299-08.
52. Zheng, B.; Zhang, Q.; Gao, J.; Han, H.; Li, M.; Zhang, J.; Qi, J.; Yan, J.; Gao, G.F. Insight into the interaction of metal ions with TroA from *Streptococcus suis*. *PLOS ONE* **2011**, *6*, e19510, doi:10.1371/journal.pone.0019510.
53. Schreur, P.J.W.; Rebel, J.M.J.; Smits, M.A.; van Putten, J.P.M.; Smith, H.E. TroA of *Streptococcus suis* is required for manganese acquisition and full virulence. *J Bacteriol* **2011**, *193*, 5073-5080, doi:10.1128/JB.05305-11.
54. Banerjee, S.; Wei, B.; Bhattacharyya-Pakrasi, M.; Pakrasi, H.B.; Smith, T.J. Structural determinants of metal specificity in the zinc transport protein ZnuA from *synechocystis* 6803. *J Mol Biol* **2003**, *333*, 1061-1069, doi:10.1016/j.jmb.2003.09.008.
55. Wei, B.; Randich, A.M.; Bhattacharyya-Pakrasi, M.; Pakrasi, H.B.; Smith, T.J. Possible regulatory role for the histidine-rich loop in the zinc transport protein, ZnuA. *Biochem.* **2007**, *46*, 8734-8743, doi:10.1021/bi700763w.
56. Kanteev, M.; Adir, N. Arginine 116 stabilizes the entrance to the metal ion-binding site of the MntC protein. *Acta Cryst. F* **2013**, *69*, 237-242, doi:10.1107/s174430911300153x.
57. Rukhman, V.; Anati, R.; Melamed-Frank, M.; Adir, N. The MntC crystal structure suggests that import of Mn^{2+} in cyanobacteria is redox controlled. *J Mol Biol* **2005**, *348*, 961-969, doi:10.1016/j.jmb.2005.03.006.
58. Bartsevich, V.V.; Pakrasi, H.B. Molecular identification of an ABC transporter complex for manganese: analysis of a cyanobacterial mutant strain impaired in the photosynthetic oxygen evolution process. *EMBO J* **1995**, *14*, 1845-1853, doi:10.1002/j.1460-2075.1995.tb07176.x.
59. Lee, Y.H.; Deka, R.K.; Norgard, M.V.; Radolf, J.D.; Hasemann, C.A. *Treponema pallidum* TroA is a periplasmic zinc-binding protein with a helical backbone. *Nat Struct Biol* **1999**, *6*, 628-633, doi:10.1038/10677.
60. Lee, Y.H.; Dorwart, M.R.; Hazlett, K.R.; Deka, R.K.; Norgard, M.V.; Radolf, J.D.; Hasemann, C.A. The crystal structure of Zn(II)-free *Treponema pallidum* TroA, a periplasmic metal-binding protein, reveals a closed conformation. *J Bacteriol* **2002**, *184*, 2300-2304, doi:10.1128/jb.184.8.2300-2304.2002.
61. Desrosiers, D.C.; Sun, Y.C.; Zaidi, A.A.; Eggers, C.H.; Cox, D.L.; Radolf, J.D. The general transition metal (Tro) and Zn^{2+} (Znu) transporters in *Treponema pallidum*: analysis of

metal specificities and expression profiles. *Mol Microbiol* **2007**, *65*, 137-152, doi:10.1111/j.1365-2958.2007.05771.x.
62. Hazlett, K.R.O.; Rusnak, F.; Kehres, D.G.; Bearden, S.W.; La Vake, C.J.; La Vake, M.E.; Maguire, M.E.; Perry, R.D.; Radolf, J.D. The *Treponema pallidum tro* operon encodes a multiple metal transporter, a zinc-dependent transcriptional repressor, and a semi-autonomously expressed phosphoglycerate mutase. *J Biol Chem* **2003**, *278*, 20687-20694.
63. Sheng, Y.; Fan, F.; Jensen, O.; Zhong, Z.; Kan, B.; Wang, H.; Zhu, J. Dual Zinc Transporter systems in *Vibrio cholerae* promote competitive advantages over gut microbiome. *Infect Immun* **2015**, *83*, 3902-3908, doi:10.1128/IAI.00447-15.
64. Radka, C.D.; Labiuk, S.L.; DeLucas, L.J.; Aller, S.G. Structures of the substrate-binding protein YfeA in apo and zinc-reconstituted holo forms. *Acta Cryst. D* **2019**, *75*, 831-840, doi:doi:10.1107/S2059798319010866.
65. Radka, C.D.; DeLucas, L.J.; Wilson, L.S.; Lawrenz, M.B.; Perry, R.D.; Aller, S.G. Crystal structure of *Yersinia pestis* virulence factor YfeA reveals two polyspecific metal-binding sites. *Acta Crystallogr D* **2017**, *73*, 557-572, doi:doi:10.1107/S2059798317006349.
66. Bearden, S.W.; Perry, R.D. The Yfe system of *Yersinia pestis* transports iron and manganese and is required for full virulence of plague. *Mol Microbiol* **1999**, *32*, 403-414, doi:10.1046/j.1365-2958.1999.01360.x.
67. Desrosiers, D.C.; Bearden, S.W.; Mier, I.; Abney, J.; Paulley, J.T.; Fetherston, J.D.; Salazar, J.C.; Radolf, J.D.; Perry, R.D. Znu is the predominant zinc importer in *Yersinia pestis* during *in vitro* growth but is not essential for virulence. *Infect Immun* **2010**, *78*, 5163-5177, doi:10.1128/IAI.00732-10.
68. Bobrov, A.G.; Kirillina, O.; Fosso, M.Y.; Fetherston, J.D.; Miller, M.C.; VanCleave, T.T.; Burlison, J.A.; Arnold, W.K.; Lawrenz, M.B.; Garneau-Tsodikova, S., et al. Zinc transporters YbtX and ZnuABC are required for the virulence of *Yersinia pestis* in bubonic and pneumonic plague in mice. *Metallomics* **2017**, *9*, 757-772, doi:10.1039/c7mt00126f.
69. El-Gebali, S.; Mistry, J.; Bateman, A.; Eddy, S.R.; Luciani, A.; Potter, S.C.; Qureshi, M.; Richardson, L.J.; Salazar, G.A.; Smart, A., et al. The Pfam protein families database in 2019. *Nucleic Acids Res* **2019**, *47*, D427-D432, doi:10.1093/nar/gky995.
